# Comparative efficacy of citric acid, Spirulina platensis, and their combination as alternatives to an antibiotic growth promoter on the performances of broilers

**DOI:** 10.5455/javar.2022.i562

**Published:** 2022-01-14

**Authors:** Jamia Ismita, Khan Md. Shaiful Islam, Mohammad Al-Mamun, Momota Rani Debi

**Affiliations:** 1 Department of Animal Nutrition, Bangladesh Agricultural University, Mymensingh 2202, Bangladesh

**Keywords:** Broiler, citric acid, spirulina, antibiotic, growth promoter, productive, performances

## Abstract

**Objective:**

A 35-day-long trial was conducted to compare the consequences of supplementing citric acid (CA),* Spirulina platensis*, and their combination as natural substitutes for antibiotic growth promoters (AGP) on the productive performance, dressing percentage, and blood serum metabolites of broilers.

**Materials and Methods:**

A total of 150-day-old Cobb-500 broiler chicks were randomly assigned to 5 dietary treatment groups, each having 30 chicks in it (10 chicks per replicate cage). The dietary groups were as follows: 1- Control (corn–soybean-based), 2- Control + antibiotic (Enrofloxacin, 10 mg/kg bodyweight), 3- Control + 1% *S. platensis*, 4- Control + 0.5% CA, and 5- Control + 1% spirulina + 0.5% CA. Starting from day 7 up to day 21, antibiotics were administered via water; spirulina and CA were offered via feed.

**Results:**

When compared to the control, all dietary supplements significantly (p < 0.05) improved broiler growth performance. However, dressing characteristics and serum metabolites (serum creatinine, albumin, and uric acid) were not influenced (*p *> 0.05) by dietary changes, except cholesterol level (*p < *0.05). Supplementation with antibiotics, CA, and spirulina produced comparable results. Among them, the CA-fed group recorded the highest live weight, weight gain, feed intake, improved feed conversion ratio, and the lowest serum cholesterol level (106.5 mg/dl). The combination group performed better than the control, but not as well as their single supplementations.

**Conclusions:**

CA and *S. platensis* might both be good candidates as natural alternatives to AGP (Enrofloxacin). But, among them, CA performed better in terms of production performance.

## Introduction

In the past 50 years, using antibiotic growth promoters (AGP) in poultry production for therapeutic or prophylactic purposes to control disease has been a common practice, which enhances profitability by improving growth and feed conversion efficiency. However, in 2006, the European Union banned feed grade AGPs, due to the possibility of cross-resistance via the food chain and the risk of possible multiple drug resistances in human pathogenic bacteria. Due to this growing concern arising among the people about food safety and general health issues, poultry nutritionists and feed manufacturers are looking for suitable alternatives to commonly used AGPs. Many scientists have already found numerous natural products like prebiotics, probiotics, organic acids, enzymes, plant extracts, herbs, herbal products, and essential oils that can be used as alternatives to AGPs safely for poultry as well as humans [1].

Citric acid (CA) is an organic acid that has been used as a natural food preservative throughout the world for many years to keep the freshness and shelf life of food products due to its inhibiting capacity of microbial action [2]. Reduced intestinal pH (3.5–4.0) caused by CA creates an acidic environment that promotes the growth of certain beneficial bacteria (Lactobacilli) while reducing or completely stopping the activity of *Escherichia coli*, *Salmonella*, and other Gram-negative bacteria [3,4]. It activates proteolytic enzymes; increases protein digestibility [5,6]; improves growth performance [6,7]; and improves calcium, phosphorus, and magnesium metabolism [8] in broilers. It improves gut health and nonspecific immunity in broiler chicks while reducing microbial load and subclinical infections [1,9,10]. These benefits make it an appropriate natural alternative for antibiotics [11] and growth promoters in poultry diets [12]. Although its supplementation increases the feed cost of broilers, increased production and feed efficiency make it cost-effective to use commercially [13].

Spirulina platensis (blue-green filamentous microalgae) possesses a tremendous nutritional profile, therapeutic properties, and several biological activities. Consequently, it has been used for many centuries as a food source for humans and animals. It is one of the richest organic nutrient sources of quality proteins (50%–70%) and contains all the essential amino acids in a balanced proportion [14,15], carbohydrates (12%–13%), fat (6%), minerals (7%), and vitamins like thiamin, riboflavin, pyridoxine, vitamin B12, and vitamin C [14,16]. In addition, it is well known to have antioxidant properties, such as phycocyanin, β-carotene, tocopherol, and xanthophyll phytopigments, and bioactive compounds, such as phycobiliproteins, phenolic acids, omega-3 and omega-6 polyunsaturated fatty acids (PUFA), phenol, gamma-linolenic acid, and flavonoids [17]. For that reason, the biologically active compounds present in it enhance immune function and growth. For that reason, it has been used as a functional feed ingredient in broilers [18]. In much of the research, it has been used as a growth promoter [14,19], a replacer of AGP [20], a gut health regulator [21], and an immunostimulator [22] in broilers. According to Bonos et al. [18], PUFAs found in spirulina increase the PUFA content of thigh meat in broilers. Phenolic, flavonoid, and antioxidant contents have a synergistic effect that improves broiler meat production [23]. Spirulina is relatively expensive to produce, purchase, and is not as widely available as other feed additives [24], even though many researchers [19,22] reported Spirulina supplementation to be cost-effective for improving broiler growth and meat quality.

Enrofloxacin, a fluoroquinolone, has been used in poultry production worldwide and its increasing resistance against Salmonella serovars, Campylobacter spp*.*, and E. coli is becoming a major public health concern in many countries, like Bangladesh, where poultry farmers use antibiotics in poultry production without following veterinary prescriptions and supervision [25,7]. Its recommended dose is 10 mg/kg of body weight/day for 3–10 days in chicken (broiler and layer) and turkey [26]. Roth et al. [7] reported that organic acid-based feed additives contributed to better growth performances than groups treated with Enrofloxacin.

The use of CA and spirulina as alternatives to AGP is not a new phenomenon yet. The problem lies in their availability and cost related to broiler production. The comparative effectiveness of CA and spirulina as alternatives to antibiotics has not been evaluated. As a result, the purpose of this study is to compare the effects of CA and *S. platensis* alone and in combination with an AGP (Enrofloxacin) on broiler growth performance, dressing percentage, and serum metabolites.

## Materials and Methods

### Experimental design and diet

In total, 150 Cobb-500-day-old unsexed broiler chicks weighing approximately 44.0 gm were reared for a period of 35 days. Based on a completely randomized design, the chicks were allotted to five dietary groups. Each group was repeated thrice, with 10 chicks per repetition. The basal diet was corn–soybean- based meal. The inclusion level used to supplement the basal diet in this trial was based on Islam [2] and Elbaz et al. [3] for CA and Sugiharto et al. [21] and Alwaleed et al. [27] for spirulina. For 7–21 days, Square Pharmaceuticals Ltd. (Bangladesh) supplied drinking water.

The dietary groups were:

Group 1- Control (corn–soybean-based) 

Group 2- Control + antibiotic (Enrofloxacin, 10 mg/kg BW)

Group 3- Control + 1% *S. platensis*

Group 4- Control + 0.5% CA 

Group 5- Control + 1% spirulina + 0.5% CA. 

### Management of birds

From beginning to end, the chicks were raised in an open-sided broiler house, and the chicks were kept in 15 cages (120 × 76 cm). Sawdust was used as litter under the cages and droppings were allowed to fall on it. Frequent cleaning of the trial house and regular observation of birds for any type of clinical sign were followed. Proper sanitary measures were taken during the experimental period. The chicks were vaccinated against Newcastle disease (ND) and infectious bursal disease according to the manufacturer’s instructions on days 4 and 11, respectively, through eye drops. Booster doses for ND were administered on day 20 into the experiment. Continuous lighting of 24 hours, ad libitum feeds (in mash form) and water were available during the whole trial time. Commonly available feed ingredients were used to formulate the diet. Table 1 presents the ingredients and analyzed composition of the diet where proximate chemicals, namely dry matter (DM), crude protein (CP), crude fiber (CF), ether extract, and ash were analyzed as recommended by AOAC [28]. Calcium and total phosphorus were analyzed according to NRC [29], and metabolizable energy (ME) was calculated based on the Bolton formula [30]. 

**Table 1.** Composition of basal diet (as dry basis) used in the study.

Ingredients	Composition (%)	Analyzed composition	Result
Maize	48.20	ME (Kcal/kg)	3,000
Rice polish	2.80	DM (%)	89.63
Soybean meal	39.00	CP (%)	22.17
Limestone	1.40	CF (%)	4.79
Mineral mixture ^b^	0.25	Calcium (%)	0.99
Di-calcium phosphate	1.55	Phosphorus (%)	0.42
Soybean oil	4.50		
Vitamin premix ^a^	0.45		
DL-Methionine	0.20		
Choline chloride	0.15		
Salt	0.50		
Starch	1.00		
Total	100		

a 4.5 gm vitamin premix: vitamin A, 13,500 IU; vitamin D_3_, 1,500 IU; α-DL-tocopherol acetate, 50 mg; menadione, 2 mg; thiamine, 3 mg; riboflavin, 5 mg; cobalamine, 15 µg; folic acid, 200 µg; nicotinic acid, 60 µg; Ca-pantothenate, 30 mg; Choline, 750 mg; ascorbic acid, 150 mg.

b 2.5 gm mineral mixture: cobalt (Co), 80 mg; copper (Cu), 2,000 mg; iodine (I), 400 mg; iron (Fe), 1,200 mg; manganese (Mn), 1,800 mg; selenium (Se), 60 mg; zinc (Zn), 14,000 mg.

### Sample collection and analysis

All birds were weighed and recorded individually after their arrival from the hatchery to the experiment farm (initial weight). Feed supply was recorded daily; body weight and feed intake (FI) were determined weekly. Each week in the morning, the birds were weighed before feed supply using an electric balance. Body weight gain (BWG), FI, and feed conversion ratio (FCR) were calculated using the following formula:

BWG = Final BW − Initial weight (gm)

FI = [Feed supplied in a week (gm) − feed weigh back in a week (gm)] / No. of bird

FCR = FI (gm) / BWG (gm)

On day 35, after weighing, three birds per dietary group were randomly selected for carcass evaluation and sampling. Viscera, gizzard, shank, and head were excised and weighed after slaughtering. Carcass weight was determined, and the dressing percentage was calculated by the following formula:

Dressing % = (Carcass weight / Live weight) × 100

To determine serum metabolites, blood samples were taken during slaughtering and centrifuged at 6,000 rpm for 10 min to separate serum. After that, the serum was stored in a properly marked Eppendorf tube at −20°C until analysis. Serum creatinine, albumin, urea, and cholesterol were analyzed in a spectrophotometer (Spectronic Genesis 5, USA) by using commercial reagent kits (LiNEAR chemicals S.L., Spain; and Human, Germany).

### Data analysis

Initially, raw data were organized in Microsoft Excel, and data were then analyzed in Statistical Package for the Social Sciences version 16. All recorded and calculated variables were analyzed by one-way analysis of variance. A statistical difference among the treatment means was determined using Tukey’s pairwise comparison test (1953) with a 5% probability.

## Results and Discussion

### Growth performances

The impacts of different dietary groups on the performance of broiler chicks are shown in Table 2. All the supplemented groups significantly improved (p < 0.05) in live weight, weight gain, FI, and FCR compared to the control on days 28 and 35. Initial weight was similar for all dietary groups. In both the study periods, the CA -supplemented group recorded significantly (*p* < 0.05) higher live weight and cumulative weight gain compared to the other supplemented groups and control. The results revealed that after the CA -supplemented group, enhanced live weight and cumulative weight gain were observed higher in the antibiotic, spirulina, and combination groups, respectively, on days 28 and 35. Significantly (*p* < 0.05) increased FI was observed in the CA -treated group in both experimental periods, followed by the antibiotic, spirulina, and combination groups, respectively. Overall, FCR was improved (*p* < 0.05) in all supplemented groups compared to the control. Broilers fed a diet with 0.5% CA supplement showed the best FCR. At the end of the trial (day 35), the spirulina, antibiotic, and combination groups had comparable FCR, which was significantly (p < 0.05) better than the control. The results are well matched with the previous outcomes [2,6,31], which narrated that diet supplementation with 0.5% CA significantly (*p* < 0.05) increases the live weight, weight gain, FI, and FCR of broilers compared with birds that had non-supplemented diets. According to Asgar et al. [32], the 0.5% CA-fed group obtained the highest live weight, average weight gain, cumulative FI, and best FCR compared to the prebiotic, antibiotic, and control groups. It was shown that the organic acid group performed better than the Enrofloxacin-treated group [7]. According to Abd-Elsamee et al. [33], CA could alleviate the negative effects of feeding a low CP diet to broilers due to its beneficial impacts on intestinal morphology. Other studies revealed that CA supplementation did not affect weight gain [8], FI [3,4,7], and FCR [34] significantly (p > 0.05), which contradicts the present study. Lower levels of CA (0.5%–3%) improve productive performance, and higher levels of CA (6%) increase sourness, and subsequently decrease feed consumption [2,34]. This may be due to mild doses of CA increasing the palatability of feed and improving FI. Dietary acidification reduces the pH values of the feed and gastrointestinal tract, which modulates the gut microbial community in a positive way [2] and reduces competition between host and microbes for nutrients, which ultimately increases nutrient availability, digestibility, and utilization [5]. Furthermore, organic acid supplementation increases the villus length [2,3,35], which increases the absorptive surface area and facilitates nutrient utilization that ultimately promotes growth performance in broilers [36].

**Table 2.** Growth performances and carcass parameters of broilers in different dietary groups.

Dietary groups							*P*-value
Parameters	Control	Enrofloxacin (10 mg/kg BW/day)		*S. platensis* (1%)	Citric acid (0.5%)	*S. platensis* (1%) + Citric acid (0.5%)	
Live weight (gm)							
Initial	44 ± 0.8	44 ± 0.9	43 ± 1.4		44 ± 0.3	44 ± 1.3	0.446
Day 28	816^b^ ± 26.4	890^ab^ ± 3.3	886^ab^ ± 20.4		933^a^ ± 8.3	862^ab^ ± 21.7	0.034
Day 35	1,256^c^ ± 39.2	1,500^a^ ± 8.8	1,475^ab^ ± 43.3		1,539^a^ ± 21.8	1,395^b^ ± 69.8	<0.001
Weight gain (gm)							
Day 1–28	768^b^ ± 79.6	845^ab^ ± 4.0	846^ab^ ± 25.1		888^a^ ± 5.61	814^ab^ ± 25.0	0.037
Day 1–35	1,207^c^ ± 42.9	1,456^ab^ ± 9.3	1,433^ab^ ± 34.6		1,494^a^ ± 18.4	1,349^b^ ± 76.8	<0.001
FI (gm)							
Day 1–28	1,330^b^ ± 55.6	1,375^ab^ ± 15.8	1,348^b^ ± 23.4		1,419^a^ ± 30.3	1,337^b^ ± 77.8	0.003
Day 1–35	23,00^c^ ± 86.5	2,498^a^ ± 15.9	2,437^ab^ ± 43.3		2,500^a^ ± 38.7	2,412^ab^ ± 55.2	0.004
FCR							
Day 1–28	1.73^a^ ± 0.11	1.63^b^ ± 0.02	1.59^b^ ± 0.04		1.60^b^ ± 0.06	1.64^b^ ± 0.24	0.039
Day 1–35	1.91^a^ ± 0.11	1.72^b^ ± 0.01	1.70^b^ ± 0.01		1.67^c^ ± 0.01	1.78^b^ ± 0.29	0.006
Carcass parameters							
Dressed wt. (gm)	870 ± 73.2	1,100 ± 74.3	1,090 ± 71.0		1,180 ± 74.1	979.3 ± 73.8	0.698
Dressing %	69 ± 0.82	73 ± 0.82	73 ± 0.82		76 ± 0.82	70 ± 0.82	0.210

^abc^Mean ± SD, means with different superscript letters within the same row differ significantly (*p *<0.05).

According to Shanmugapriya et al. [37], Abdel Moneim et al. [38], and Atiyah and Hamood [39], 1% spirulina supplementation can improve weight gain and FCR significantly (p < 0.05). This can be attributed to spirulina supplementation improving dietary vitamin and mineral absorption and enhancing gut integrity and the immune system [22]. Furthermore, the physiological functions of the bioactive components present in spirulina may be involved in the positive effect on growth performance.

### Dressing characteristics

Dietary supplements failed to affect (p > 0.05) dressing characteristics and are shown in Table 2. Numerically, the highest dressed weight and dressing percentage were observed in the CA-fed group, followed by the antibiotic, spirulina, and combination groups, and the lowest in control. It was observed that a study was conducted under heat stress conditions on broilers and the authors reported an insignificant (p > 0.05) dressing percentage for dietary supplementation of 0.5% CA in single and in combination with probiotics and garlic [3], which agrees with the results of this study. Also, Sabour et al. [4] observed that CA containing an organic acid blend failed to affect (p > 0.05) dressing parameters in broilers, which is also in favor of this present results. But the findings of Chowdhury et al. [40] contradict the current outcomes and state that 0.5% CA alone or in combination with an antibiotic significantly (*p* < 0.05) improved dressed weight in broilers. Alwaleed et al. [27], Khan et al. [22], and Abdel-Moneim et al. [38] reported that spirulina supplementation had a significant (*p* < 0.05) effect on dressing percentage, which also contradicts the current results. Rubel et al. [20] observed that the supplementation of S. platensis against an antibiotic failed to affect (p > 0.05) dressing percentage in broilers, which agrees with the present findings. The numerically higher dressing percentage of this study may be due to higher live weight, which is correlated to a higher dressing percentage and vice versa.

**Table 3.** Blood parameters of birds receiving different dietary groups.

	Dietary groups					*p*-value
Parameters	Control	Enrofloxacin (10 mg/kg BW/day)	*S. platensis* (1%)	Citric acid (0.5%)	*S. platensis* (1%) + Citric acid (0.5%)	
Creatinine (mg/dl)	0.104 ± 0.04	0.15 ± 0.03	0.55 ± 0.59	0.17 ± 0.03	0.23 ± 0.06	0.530
Albumin (gm/dl)	1.04 ± 0.37	0.58 ± 0.05	0.53 ± 0.35	0.91 ± 0.07	0.62 ± 0.68	0.622
Urea (mg/dl)	5.79 ± 1.94	3.42 ± 0.40	4.3 ± 1.44	7.74 ± 0.58	6.63 ± 1.6	0.103
Cholesterol (mg/dl)	139.6^a^ ± 5.5	109^b^ ± 26.2	107.4^b^ ± 4.2	106.5^b^ ± 8.6	114^ab^ ± 23	0.037

^abc^Mean ± SD, means with different superscript letters within the same row differ significantly (*p *< 0.05).

### Serum metabolites

Serum metabolites of different dietary groups of broilers are presented in Table 3. Except for cholesterol level (*p* < 0.05), other parameters (serum creatinine, uric acid, and albumin) were not significantly (p > 0.05) affected by dietary changes. Apart from the combination group, serum cholesterol was significantly lower (*p* < 0.05) in all dietary groups than control. This result agrees with the findings of Elbaz et al. [3] and Abdel Fattah et al. [10], who stated that CA supplementation in the diet significantly (*p* < 0.05) reduced serum cholesterol levels, while serum albumin and uric acid levels were not affected (p > 0.05) among the groups. But this result contradicts the findings of Abd-Elsamee et al. [33], who observed significant (*p* < 0.05) improvement in serum cholesterol, albumin, and uric acid levels in broilers when a low CP diet was fed.

On the other hand, 1% S. platensis also considerably (*p* < 0.05) decreased serum cholesterol and triglyceride levels in heat-stressed broilers, and serum albumin, creatinine, and uric acid did not show significant (p > 0.05) differences [27,38]. This supports the findings of the present study. The hypocholesterolemic effects of CA supplementation can be described as CA can deconjugate and dehydroxylate bile acids that inhibit lipid absorption and fatty acid synthesis. As a result, fecal excretion of fatty acids increases and ultimately lowers blood lipid profiles [3]. Furthermore, dietary supplementation that contains photogenic compounds reduces blood cholesterol levels [41]. The antioxidant properties of spirulina, such as phycocyanin, linolenic acid, and phenolic compounds, may be attributed to the decreased serum cholesterol and triglyceride levels. The C-phycocyanine protein of spirulina inhibits pancreatic lipase activity in a dose-related manner, which is responsible for the hypocholesterolemic effect [42].

From the above results, it is clear that both CA and spirulina have their own specialty to improve production in broiler. But the limitation is that spirulina is not as available as CA in the local market which makes it little more expensive. Its availability in the local market will broaden the scope of using this superior alga in sustainable poultry production.

## Conclusion

This study revealed that dietary supplementations of 0.5% CA and 1% spirulina improved growth performance and lowered serum cholesterol levels in broiler chickens. Both have enough potential to replace the AGP (Enrofloxacin). Among them, our study suggests using CA in broiler production due to its availability as compared to spirulina. The combination group provided better results than the control but not as satisfactory as their single inclusion in the diet. Therefore, further research should be carried out to explore their single and combined effects to improve broiler production at the lowest economic cost.

## List of Abbreviations

AGP, antibiotic growth promoter; BW, body weight; CA, citric acid; dl, deciliter; FCR, feed conversion ratio; FI, feed intake; IU, international unit; Mcal, mega calorie; ME, metabolizable energy; mg, milligram; rpm, rotation per minute; SD, standard deviation; wt., weight; µg, microgram.
